# Encapsulated neoplasms of the thyroid gland

**DOI:** 10.1007/s00428-025-04375-0

**Published:** 2025-12-18

**Authors:** Nikolina Dioufa, Zubair W. Baloch

**Affiliations:** Department of Pathology & Laboratory Medicine, Perelman School of Medicine, University of Pennsylvania, Philadelphia, PA USA

**Keywords:** Thyroid gland, Neoplasms, Carcinoma, Encapsulated, Adenoma, Low-risk neoplasms, Carcinoma

## Abstract

Encapsulated thyroid gland lesions, defined by complete or partial confinement within a fibrous capsule, are common findings in endocrine pathology but frequently pose diagnostic challenges. The primary difficulty lies in distinguishing benign, low-risk, and malignant neoplasms, particularly within the spectrum of follicular-patterned tumors. Accurate classification can be hindered by pitfalls such as differentiating true tumor capsule from peritumoral fibrosis, identifying capsular or vascular invasion versus reactive changes from preoperative fine-needle aspiration, and accounting for histologic and cytologic heterogeneity. In this review, we discuss the definition of true capsule and vascular invasion and how to contrast from mimics. We describe the wide spectrum of both follicular and non-follicular lesions encountered in the thyroid, and we propose a systematic diagnostic approach to encapsulated thyroid neoplasms, integrating ultrasonographic, cytologic, histologic, immunohistochemical, and molecular data, in an effort to optimize diagnostic accuracy and guide appropriate clinical management.

## Introduction

Encapsulated thyroid gland lesions are numerous neoplasms that are entirely or partially contained by a variably thick fibrous layer. They are quite common in clinical practice and can pose diagnostic challenges, mainly differentiating between benign, low-risk, and malignant encapsulated thyroid neoplasms. Usually, encapsulation portends an indolent clinical behavior as compared to tumors with infiltrative growth. Accurate classification of an encapsulated thyroid neoplasm remains a significant source of uncertainty among pathologists, generalists, as well as experienced endocrine pathologists [1, 2] The diagnostic conundrums associated with an encapsulated thyroid neoplasm include differentiating true tumor capsule from peritumoral fibrosis, invasion of the tumor capsule or angioinvasion from reactive and reparative changes due to preoperative fine-needle aspiration, and lastly, the heterogeneity of growth patterns and cytologic features, allowing for accurate classification.

The most significant accomplishment in the classification of the encapsulated thyroid neoplasms was the renaming of the non-invasive encapsulated follicular variant of papillary thyroid carcinoma (non-invasive FVPTC) to noninvasive follicular thyroid neoplasm with papillary-like nuclear features (NIFTP) in 2016, to reduce overtreatment of this neoplasm with no to low risk of recurrence. Though often discussed, the introduction of NIFTP was instrumental in bringing to light challenges and subjectivity in assessing capsular and vascular invasion in an encapsulated thyroid neoplasm, leading to significant interobserver variability and impacting patient management [3, 4].

Fine-needle aspiration (FNA) has proven to be essential for thyroid nodule evaluation. It has been shown that most encapsulated thyroid neoplasms, especially those with follicular-patterned growth, are diagnosed as indeterminate for malignancy on FNA. Molecular profiling of these lesions has shown that most harbor *RAS* or *RAS*-like mutations.

In this review, we outline a systematic approach for diagnosing encapsulated thyroid lesions, with a particular emphasis on the most encountered follicular-patterned neoplasms. We aim to construct a practical diagnostic overview and framework by integrating ultrasonographic, cytologic, histologic, immunohistochemical, and molecular findings.

## Tumor capsule: definition, features, and mimics

While conceptually perceived as simple, a true definition of a tumor *capsule* in thyroid pathology does not exist. The encapsulated lesions are usually confined by a rim of fibrous tissue of variable thickness. Thin capsules are mostly associated with adenomas and are imperceivable in cases of adenomatous/hyperplastic nodules. A thick tumor capsule is frequently seen in carcinomas and warrants a thorough examination.

Adding to the complexity, not all capsules are created equal. Some tumors are partially encapsulated or surrounded by dense fibrosis that may or may not represent a true capsule. Changes from prior fine-needle aspiration (FNA),including hemorrhage, granulation tissue, and scarring,can further obscure the interface between tumor and capsule, making it harder to distinguish real invasion from post-biopsy artifacts.

## Tumor capsule invasion: criteria, mimics, and controversies

Determining whether a thyroid tumor shows capsular invasion has been central to distinguishing benign from malignant and borderline entities. Yet this determination remains one of thyroid pathology’s most diagnostically challenging aspects, and likely stems from interpretive subjectivity and technical limitations in tissue processing and sampling.

**Fig. 1:**
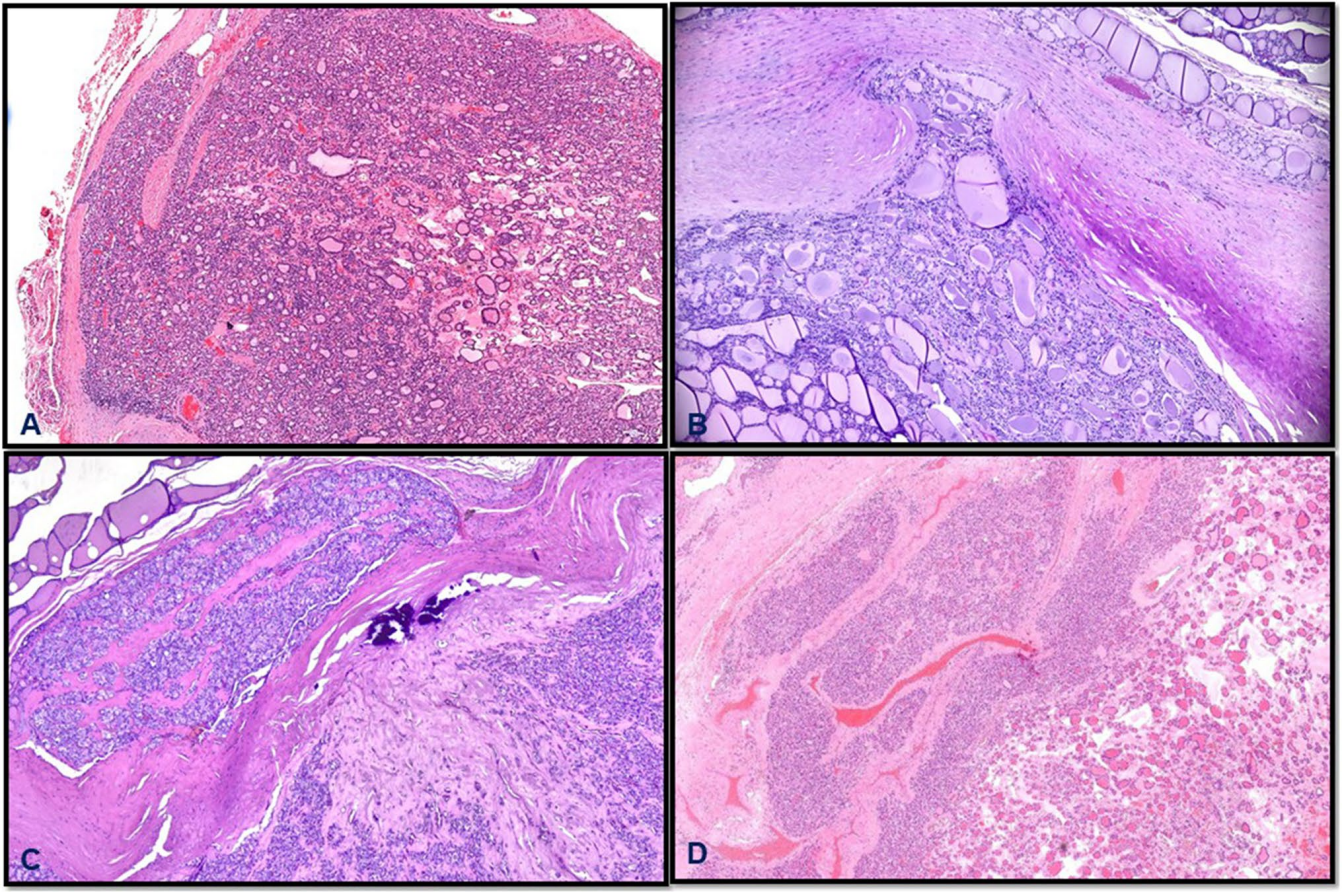
Classic examples of capsular invasion. Trans-capsular invasion/“mushrooming” of the tumor (**A**, **B**). Separate tumor foci seen within and beyond the tumor capsule (**C**, **D**)

According to the World Health Organization (5th edition, 2022) and the College of American Pathologists (CAP) Endocrine Tumor Protocol, capsular invasion is defined as “complete penetration of the tumor capsule by neoplastic cells into the surrounding thyroid tissue or soft tissue beyond the capsule.” By contrast, partial capsular penetration does not meet the threshold for malignancy. Figure 1 illustrates classic examples of capsular invasion.

**Fig. 2:**
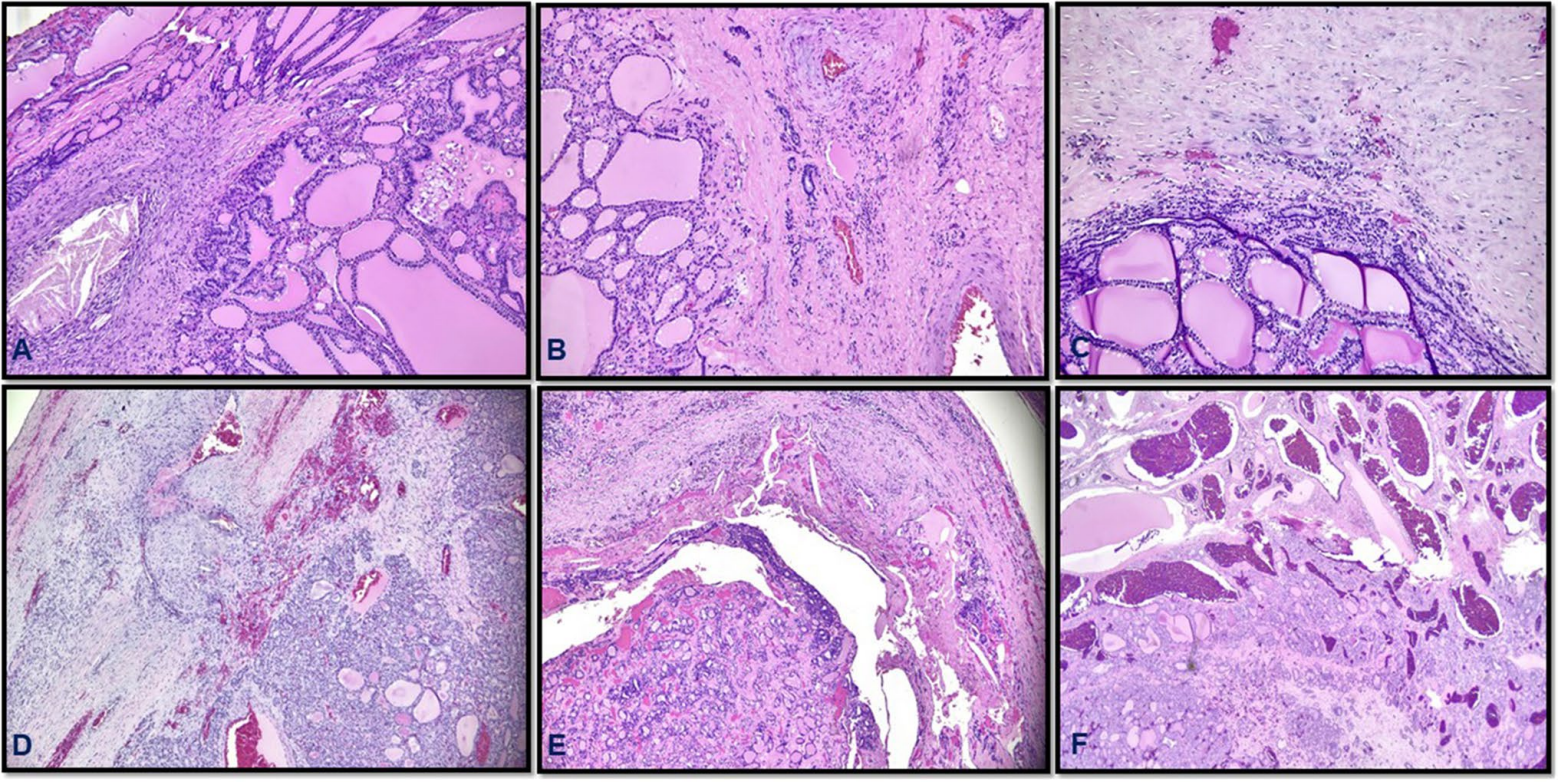
**A**–**F** Example cases of mimickers of capsular invasion in daily practice due to reactive and reparative change following preoperative fine-needle aspiration, with granulation tissue formation, vascular proliferation, and fibrosis often mimicking capsular invasion, creating diagnostic pitfalls

The real diagnostic challenge lies in differentiating true tumor capsule invasion from pseudo-invasion or sectioning artifacts (Fig. 2). Encapsulated adenomatous nodules and follicular adenomas, for instance, may have lobulated or irregular outlines that push into the capsule, especially when the capsule is thin or the tissue has been sectioned in an oblique fashion. Similarly, the nodules arising in a background of chronic lymphocytic thyroiditis may have irregular periphery due to fibrosis and inflammation in the surrounding thyroid parenchyma. These areas can closely mimic full-thickness invasion, particularly if only seen in a single focus. In contrast, actual invasion tends to show irregular or tongue-like projections of tumor cells that break through and extend beyond the outer margin of the capsule, often accompanied by stromal reaction, hemorrhage, or a so-called “mushrooming” appearance [4]. Still, even without these features, complete transgression of the capsule may be sufficient for diagnosing carcinoma [5].

This diagnostic problem is significant since misinterpreting subtle or equivocal findings as invasion risks overdiagnosis and possibly overtreatment. As a result, many experts advocate for a conservative diagnostic threshold. When invasion is suspected but not clearly demonstrated, it is appropriate to classify the case as “indeterminate for invasion.”

To reduce the risk of under- or overdiagnosis, thoroughly sampling the tumor–capsule interface is critical. Best practices recommend submitting the entire tumor capsule, with histologic sections taken at least every 2–3 mm.  Despite this approach, focal invasion may still go undetected if it is not included in the sampled sections [3]. The presence of vascular invasion can provide additional evidence in favor of malignancy.

Diagnosing capsular invasion requires a careful, methodical approach that combines meticulous grossing, comprehensive sampling, awareness of common artifacts, and strict adherence to established criteria. Given the significant clinical implications, from conservative follow-up for benign or borderline lesions to total thyroidectomy and radioactive iodine for carcinoma, it is a task that requires both precision and caution. Until we have more objective biomarkers for invasion, the diagnosis will continue to rest on scrutiny of histologic details by experienced eyes. Table 1 provides a grossing protocol for optimal assessment of capsular and vascular invasion. Whole Block Imaging (WBI) by micro-CT scanner has been suggested as a novel noninvasive imaging technique with high resolution to enhance the detection of capsular invasion in encapsulated thyroid carcinoma, identifying additional foci of capsular invasion missed by the initial H&E without tissue exhaustion, acting like virtual recuts [6]. The role of AI in this tedious task is one that remains to be further explored [7].

**Table 1:** Recommended protocol for histologic examination of thyroid nodules

1. Orientation and measurement: Record the overall dimensions of the thyroid lobe(s) and isthmus, as well as the size, location, and distance of each nodule from the capsule and surgical margins
2. Capsule assessment: Note the capsule’s presence, thickness, and integrity around each nodule. Encapsulation versus infiltrative borders should be described in detail
3. Sectioning strategy:
o Encapsulated nodules: Submit the entire tumor-capsule-adjacent parenchyma interface. This is critical to assess for capsular and vascular invasion
o The general recommendation, particularly for encapsulated follicular-patterned lesions, is to submit the entire capsule, with one histologic section per 2–3 mm capsule
o Be mindful not to overfill the cassette as this can lead to insufficient fixation and artifactual compression and protrusion of the lesion through the capsule, particularly at the edge of the section
o Non-encapsulated nodules: Representative sections from every morphologically distinct nodule should be submitted
o Tumors ≥1 cm: Entirely submit or extensively sample the tumor if it is suspicious for invasion
o Tumors < 1 cm: Submit entirely
4. Multifocality and background: Sample the surrounding thyroid parenchyma to assess for chronic lymphocytic thyroiditis, other incidental lesions, or additional tumor foci

## Angioinvasion: definition, features, and mimics

Angioinvasion, also termed as vascular invasion, is agreed upon by all as a definite criterion of malignancy in an encapsulated thyroid lesion. It also plays a major role in predicting the tumor’s metastatic potential and overall prognosis. Angioinvasion can be seen involving the blood vessels located within or outside the tumor capsule [8]. However, recognizing true vascular invasion can be notoriously tricky. It requires detailed histologic inspection, a good understanding of everyday artifacts, and adherence to the established diagnostic criteria.

**Fig. 3:**
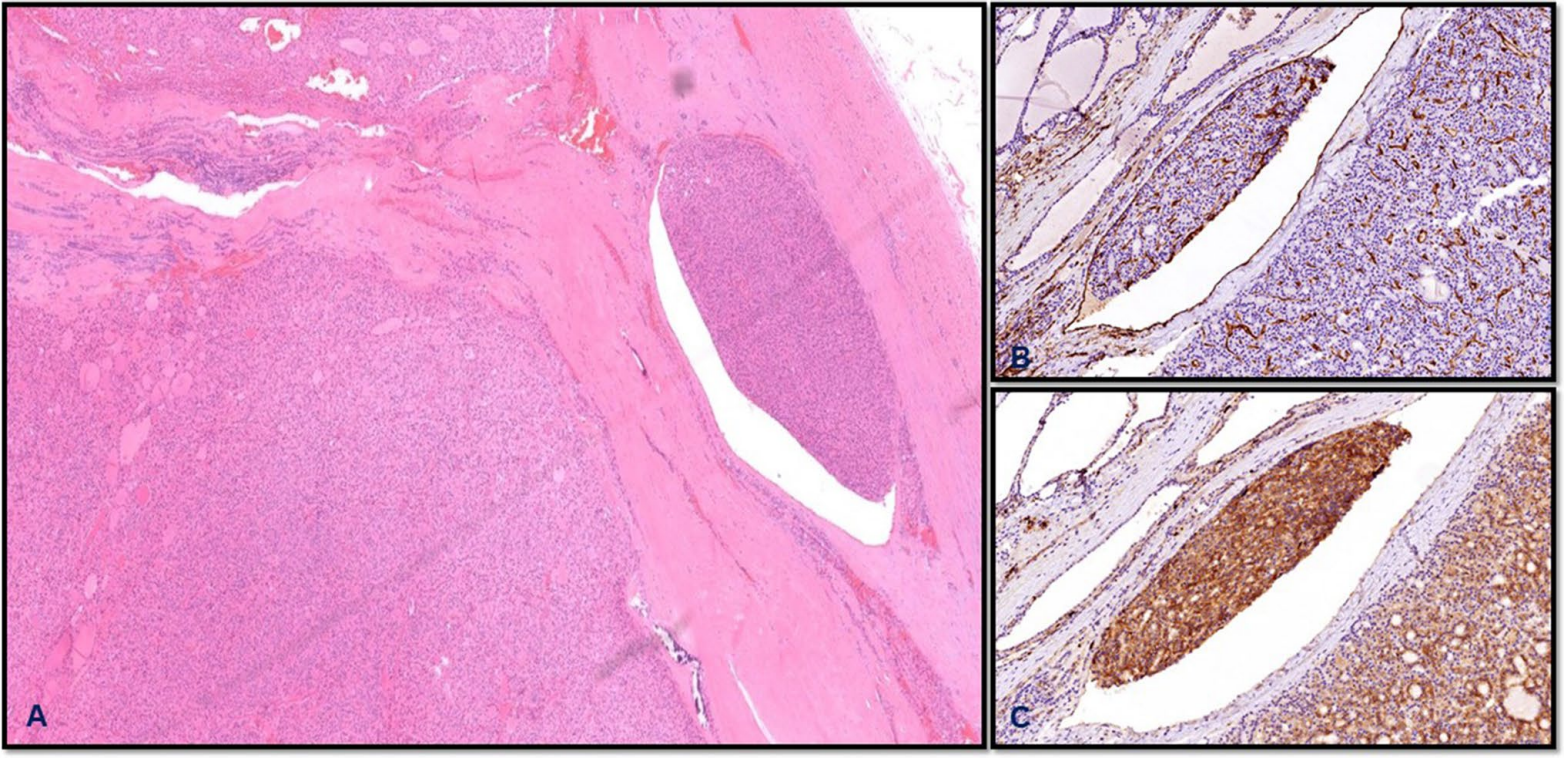
Angioinvasion. The tumor adheres to the vessel wall, partially occluding the lumen (**A**). A layer of endothelial cells enveloping the tumor thrombus, highlighted by CD31 (**B**). CD61 highlights the fibrin that is intimately admixed with the tumor cells (**C**)

According to the World Health Organization (5th edition, 2022), vascular invasion is defined as “tumor cells inside a blood vessel (excluding lymphatics) that have breached the vessel wall.” The tumor cells are typically attached to the vessel wall and are often enveloped by an endothelial lining. The College of American Pathologists (CAP) also endorses these criteria and recommends that the tumor thrombus should partially fill the vascular space (Fig. 3). However, the reliance on strict morphologic criteria for diagnosing vascular invasion can indeed raise concerns about the potential for underdiagnosis. While these criteria are designed to enhance diagnostic accuracy by minimizing false positives, they may overlook subtle (small tumor emboli) or atypical manifestations of vascular invasion that do not fit neatly within established guidelines.

The diagnostic conundrums lie in distinguishing foci of true angioinvasion from its mimics, especially in encapsulated tumors. These include tangential sections, which distort the appearance of capsular vessels, making benign findings appear suspicious. Retraction artifacts, hemorrhage from prior FNA, and poorly preserved tissue can further blur the lines [9]. Sometimes, tumor cells are seen in what appears to be a vessel. Still, they are not attached to the wall or surrounded by endothelium, features necessary for a confident diagnosis. CAP recommends a conservative approach in these situations, warning that overcalling vascular invasion can lead to unnecessary treatment, while following strict criteria is more closely tied to actual clinical outcomes.

There is an ongoing debate about the clinical significance of the extent of angioinvasion. While the presence of even a single focus is sufficient to diagnose carcinoma, emerging evidence suggests that cases with four or more foci of vascular invasion tend to behave more aggressively and carry a higher risk of metastasis. To aid clinical decision-making, some pathologists distinguish between “minimal/focal angioinvasion” (1–3 foci) and “extensive/multifocal angioinvasion” (≥ 4 foci), though these thresholds are not universally accepted [10–12]. Regardless of the terminology, a thorough sampling of the tumor-capsule interface is crucial. Blood vessels both within and beyond the tumor capsule should be examined under high magnification, looking for definite signs of vascular invasion, includingtumor adherence, fibrin thrombi, and endothelial enveloping of tumor thrombus. Immunohistochemical staining with endothelial markers such as CD31 or ERG and fibrin, like CD61, can be helpful in challenging cases, but morphology remains the gold standard [8, 12].

The clinical implications are significant. Minimally invasive follicular carcinomas, i.e., without vascular invasion, usually have an excellent prognosis and can often be managed conservatively. However, once angioinvasion is identified, especially multifocal, treatment often escalates, leading to completion thyroidectomy and radioactive iodine ablation due to the increased risk of hematogenous spread to distant sites like the lungs and bones [13].

## Preoperative management of thyroid nodules

The modern era of thyroid nodule management includes ultrasound imaging, fine-needle aspiration, and, when available, molecular testing. *Ultrasound* (US) is essential in the initial assessment of palpable and non-palpable thyroid nodules, including the encapsulated lesions. However, its ability to reliably distinguish benign from malignant encapsulated thyroid neoplasms is limited. Follicular-patterned neoplasms such as follicular adenomas, follicular carcinomas, and NIFTPs exhibit overlapping sonographic features. They are usually solid, well-circumscribed, and hypoechoic to isoechoic, often with a thin peripheral hypoechoic halo corresponding to the fibrous capsule, compressed vessels or surrounding thyroid tissue [14]. Internal vascularity may be seen, typically perinodular, but does not reliably distinguish benign from malignant neoplasms. The ultrasound features associated with increased risk of malignancy in an encapsulated thyroid neoplasm include hypoechogenicity, tumor capsule disruption with involvement of the surrounding thyroid parenchyma or extrathyroidal extension, disrupted calcifications within the tumor capsule, and increased intranodular vascularity. Importantly, these lesions often lack the high-risk US features typically associated with papillary thyroid carcinoma, such as microcalcifications, irregular margins, or a taller-than-wide shape. Elastography and contrast-enhanced ultrasound have shown potential in assessing capsule integrity and internal vascularity but are not yet standard in clinical practice [15]. This bland sonographic appearance often leads to classification as indeterminate, necessitating preoperative biopsy.

Cytologically, on FNA, the encapsulated follicular patterned lesions yield cellular aspirates composed of microfollicular or trabecular architecture, scant colloid, and uniform follicular cells,features shared across both benign and malignant entities. As a result, cytology alone is insufficient to make a definitive diagnosis, and these are frequently classified as indeterminate (Bethesda category III or IV) on FNA [16–19].

The adjunct molecular profiling of thyroid FNA specimens classified as indeterminate for malignancy can offer some diagnostic guidance. Encapsulated follicular-patterned lesions exhibit a molecular landscape distinct from infiltrative tumors. *RAS* mutations are the most frequently encountered alterations across follicular adenomas, NIFTPs, follicular carcinomas, and invasive encapsulated follicular variant of papillary carcinoma, suggesting a shared origin and early role in tumorigenesis. These alterations do not diagnose malignancy but suggest a follicular-patterned neoplasm with more indolent clinical behavior [18, 20].

The *PAX8::PPARG* gene fusion, typically associated with follicular carcinoma, results from a t(2;3)(q13;p25) translocation and is present in approximately 30–40% of follicular carcinomas [21]. It may also be seen in adenomas but is more often associated with tumors exhibiting vascular invasion. *THADA* fusions are frequently identified in follicular adenomas and are thought to represent low-risk alterations [22, 23]. *EIF1AX* mutations, while rare, may indicate a more aggressive phenotype, particularly when co-occurring with *RAS* mutations [24]. Non-V600E *BRAF* mutations, such as *BRAF* K601E, may be seen in NIFTP and are considered to confer intermediate behavior. In contrast, *TERT* promoter mutations strongly correlate with aggressive clinical behavior and are typically found in widely invasive follicular carcinomas [25]. Nevertheless, molecular findings should be interpreted with caution and in conjunction with histologic features.

## Benign and low-risk encapsulated thyroid neoplasms

Benign encapsulated thyroid lesions represent a diverse group of nodules. This group includes *adenomatous (colloid) nodules—*often seen in the setting of multinodular goiter, along with *follicular adenomas*, *oncocytic adenomas (previously known as Hürthle cell adenomas)*, and the less common *hyalinizing trabecular tumors (HTTs). Low-risk encapsulated follicular-patterned thyroid tumors include noninvasive follicular thyroid neoplasm with papillary-like nuclear features (NIFTP), minimally invasive, follicular and oncocytic carcinomas, and  invasive encapsulated follicular variant of papillary thyroid carcinoma (IEFVPTC), both minimally invasive and encapsulated angioinvasive*. These tumors are clinically important because, although they share similar appearances on imaging and cytology, however, their underlying behavior and potential for recurrence differ.

On ultrasound, these lesions tend to be well-demarcated and solid. Adenomatous nodules typically appear isoechoic to hyperechoic and often show peripheral vascularity and comet-tail artifacts due to the presence of dense colloid. Follicular adenomas are usually solid, hypoechoic nodules with a smooth border and sometimes a thin peripheral halo. Oncocytic adenomas may resemble follicular adenomas in shape and border, but often appear more hypoechoic and demonstrate increased vascularity [14]. Hyalinizing trabecular tumors can be particularly deceptive, as their solid nature and internal echogenic foci may mimic the microcalcifications seen in papillary thyroid carcinoma (PTC) on imaging [26].

Fine-needle aspiration (FNA) cytology is often inconclusive for these encapsulated lesions, leading to indeterminate diagnoses as per Bethesda classification of reporting thyroid cytology (TBSRTC). Adenomatous nodules typically yield abundant thin colloid and benign follicular cells arranged in monolayer sheets or microfollicles. Follicular adenomas produce moderately cellular aspirates, with microfollicular patterns, and minimal colloid. These features commonly result in a TBSRTC category III (Atypia of Undetermined Significance) or IV (Follicular Neoplasm) diagnosis, mainly depending on the cellularity of the specimen and the presence of microfollicles [16]. Oncocytic adenomas yield a oncocytic cell-rich aspirate with round, centrally placed nuclei, prominent nucleoli, and granular cytoplasm, often resulting in a “oncocytic neoplasm” diagnosis. HTTs can show nuclear grooves and pseudo-inclusions, which resemble those of papillary thyroid carcinoma, posing a diagnostic pitfall during cytologic interpretation and classification of the lesion as TBSRTC category V (suspicious for papillary thyroid carcinoma) [27].

By molecular profiling, the encapsulated follicular patterned lesions tend to have low-risk profiles. Follicular and oncocytic adenomas may harbor *RAS* mutations or *PAX8::PPARG* rearrangements, but these also occur in follicular carcinomas and EFV-PTC. *THADA* gene fusions, which have been described in follicular adenomas, are usually considered benign [22]. Hyalinizing trabecular tumors often show rearrangements involving the *GLIS3* gene, typically via fusion with *PAX8*, an important thyroid transcription factor. *RET/PTC* rearrangements,particularly *RET/PTC1* have been described in HTT, which adds another layer of complexity, as this genetic alteration is also found in classical PTC [27]. Additionally, NIFTP, minimally invasive and angioinvasive EFV-PTC, and follicular carcinoma are typically driven by *RAS*-like mutations, namely *NRAS* and *HRAS*, as opposed to the *BRAF* V600E mutation commonly seen in classical PTC. These molecular signatures support the biological separation of NIFTP and minimally invasive EFV-PTC from more aggressive thyroid carcinomas and align with their favorable clinical outcomes [28–30].

**Fig. 4:**
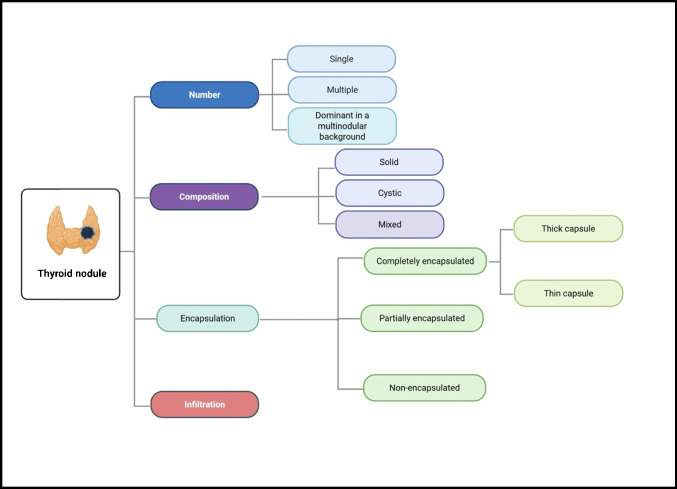
Stepwise microscopic assessment of thyroid lesions under low magnification, highlighting essential features for diagnostic interpretation (image created using Biorender.com)

**Fig. 5:**
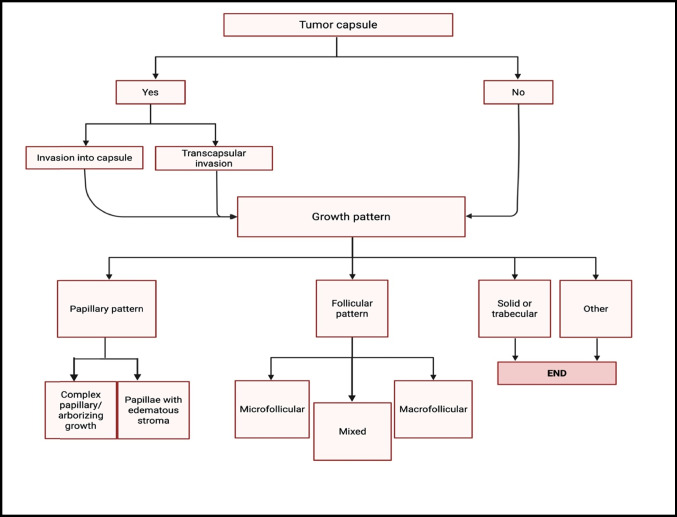
Stepwise diagnostic approach depicting the microscopic criteria for assessing capsule integrity and growth behavior in encapsulated thyroid neoplasms (image created using Biorender.com)

 The histologic evaluation commences on screening under low magnification, examining the number of thyroid nodules present, the composition, and encapsulation versus infiltrative growth (Fig. 4). The next step includes the assessment of the tumor capsule and identification of the predominant growth pattern (Fig. 5).

**Fig. 6:**
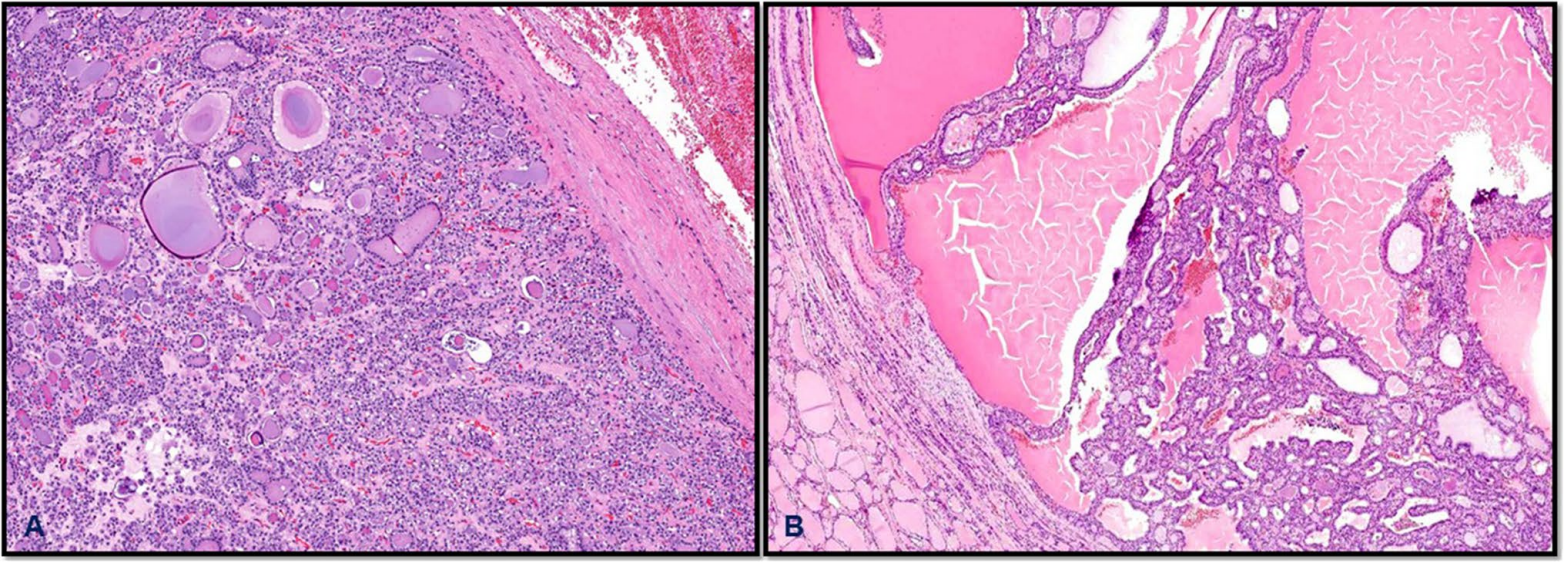
Follicular adenoma, encapsulated follicular patterned, lacking invasive features and the nuclear features of PTC (**A**). Follicular adenoma with papillary architecture, an encapsulated neoplasm showing complex papillary infoldings of the lining epithelium, with broad papillae containing embedded follicles (**B**)

Adenomatous nodules are usually poorly encapsulated and are made up of variably sized follicles rich in colloid. Follicular adenomas, in contrast, are typically well-circumscribed or encapsulated and consist of uniform follicular cells arranged in microfollicular, macrofollicular, or trabecular patterns without any evidence of capsular or vascular invasion (Fig. 6). Similarly, oncocytic adenomas are defined by their dense population of large oncocytic cells with granular eosinophilic cytoplasm (comprising > 75% of the tumor), arranged in microfollicles or trabeculae. Hyalinizing trabecular tumors, though uncommon, are usually encapsulated, with a distinctive trabecular architecture, intra-trabecular hyaline material, and low mitotic activity. Despite their cytologic resemblance to PTC, they typically lack true papillae and the fully developed nuclear features that define classical papillary carcinoma. When suspected, immunohistochemical stain with Ki67 (MiB1 clone, performed at room temperature) will give a unique membranous staining pattern that is pathognomonic for these lesions [27]. Automated platforms currently used for immunohistochemistry in routine laboratory practice may produce variable staining intensities and, at times, false-negative results. Therefore, immunostaining outcomes should be interpreted with caution, as a negative result does not necessarily exclude the diagnosis [31].

**Fig. 7:**
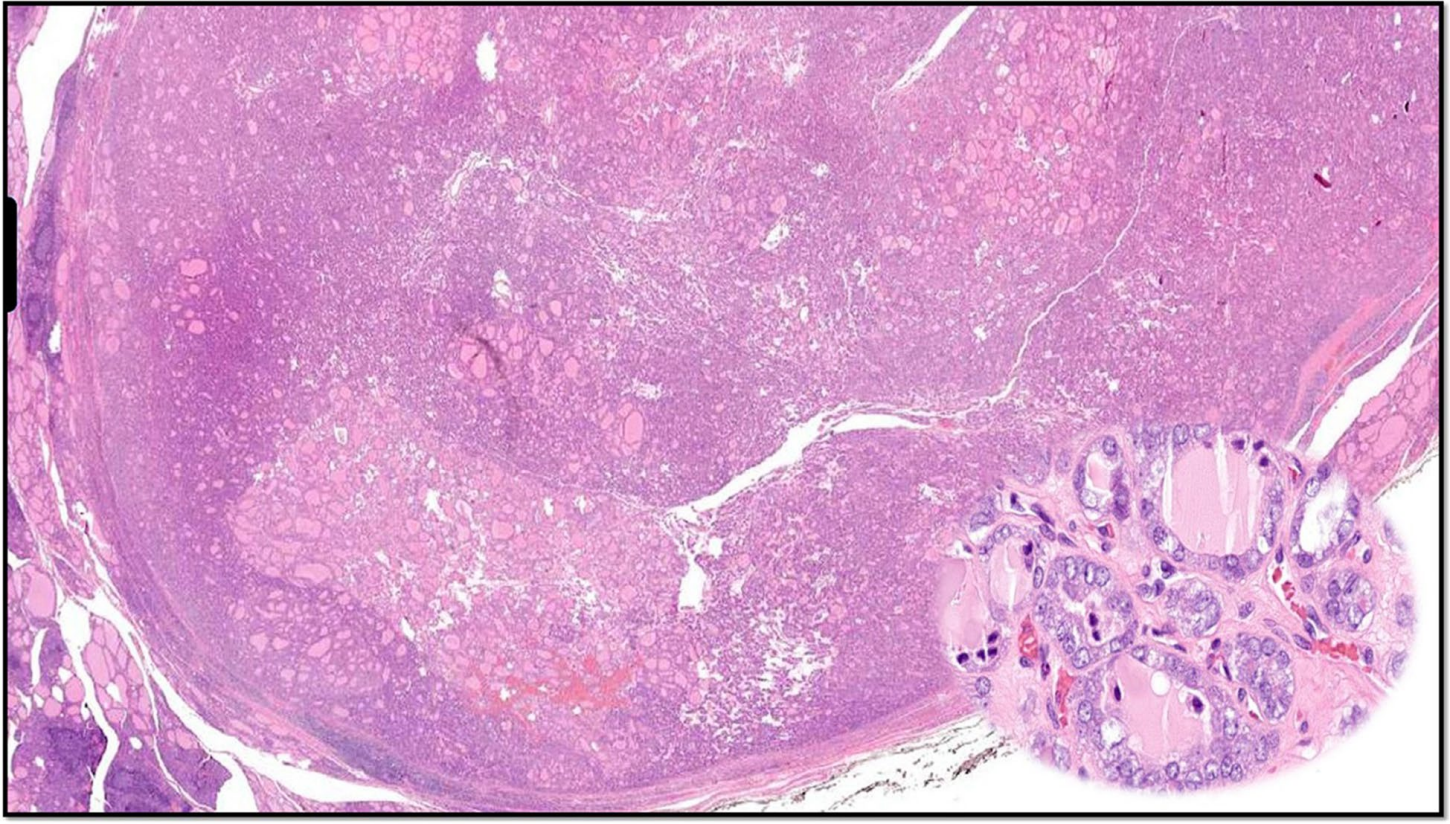
Non-invasive follicular thyroid neoplasm with papillary-like nuclear features (NIFTP). Well-demarcated lesion with follicular architecture and nuclear features of PTC (zoomed in insert)

By definition NIFTP diagnosis requires: encapsulation or clear demarcation and follicular growth pattern with all of the following: no true papillae and psammoma bodies, and < 30% solid/trabecular/insular growth pattern, nuclear features of papillary carcinoma, no invasive features (vascular or capsular invasion), no tumor necrosis and low mitotic count (< 3 mitoses/2 mm2) [32–34]. The presence of the *BRAF* V600E mutation rules out NIFTP (Fig. 7). NIFTP is considered an extremely indolent neoplasm if completely resected. A compilation of studies including > 1500 NIFTP cases reveals a rate of adverse oncological events < 1% and no reported mortality [1, 32, 33, 35–37].

Rare cases of intrathyroidal parathyroid adenoma with a prominent follicular pattern can pose a diagnostic challenge. The PTH and thyroglobulin immunostains can be particularly helpful in these cases. Additionally, intrathyroidal or ectopic thymomas can rarely be found within or attached to the thyroid (left lobe, lower pole), arising from thymic or branchial pouch remnants displaced during embryological development. The tumor is encapsulated and composed of polygonal epithelioid cells, forming lobules separated by thin to variably sclerosed or thickened fibrous septa [38].

Follicular adenoma with papillary architecture is a benign thyroid tumor that is encapsulated and displays papillary architecture. These papillae can be simple or complex, usually point to the center of the lesion, and lack the distinctive nuclear features seen in PTC. The tumor cells are uniform with even nuclear chromatin and show no signs of capsular or vascular invasion (Fig. 6). At the  molecular level, these adenomas frequently harbor mutations in either *TSHR*, *EZH1*, or *GNAS* genes and lack  *BRAF* V600E or *BRAF-like mutations*. Clinically, most of these tumors are autonomously functioning nodules presenting at a younger age and can present with symptoms of hyperthyroidism [39, 40].

## Thyroid tumors of uncertain malignant potential

Thyroid tumors of uncertain malignant potential (UMP) occupy a diagnostic gray zone in thyroid pathology; an intermediate category where lesions raise concern but do not meet the definitive criteria for malignancy. These tumors are typically encapsulated or well-circumscribed and exhibit a follicular growth pattern. While they may display worrisome focal features, such as mild nuclear atypia or subtle capsular irregularities, they fall short of the histologic thresholds required for a diagnosis of carcinoma. The UMP classification underscores the limitations of histopathologic interpretation, especially in cases complicated by technical artifacts, limited tissue sampling, or ambiguous morphologic features [41].

In its 5th edition classification (2022), the World Health Organization (WHO) identifies two main subtypes within the category of tumors of uncertain malignant potential: *follicular tumor of uncertain malignant potential (FT-UMP) and well-differentiated tumor of uncertain malignant potential (WDT-UMP) *[41, 42]. *FT-UMP* refers to an encapsulated tumor with a follicular growth pattern that closely resembles a follicular adenoma but raises concern due to focal areas where the capsule appears irregular or partially penetrated;changes suggestive, but not diagnostic, of capsular invasion. Crucially, there must be no evidence of vascular invasion, and the tumor must lack the nuclear features characteristic of papillary thyroid carcinoma (PTC) [34]. *WDT-UMP*, by contrast, shows some nuclear features associated with PTC, such as nuclear enlargement, chromatin clearing, or grooves, but these changes are insufficient in extent or severity to support a diagnosis of the   IEFVPTC. This designation may also apply to tumors that resemble noninvasive follicular thyroid neoplasm with papillary-like nuclear features (NIFTP) but fall short of meeting all diagnostic criteria, often due to subtle findings that raise concern, such as questionable papillary structures or increased architectural complexity [42].

A diagnosis of UMP usually arises when histologic interpretation is compromised by suboptimal tissue quality or sampling limitations. A common scenario is a thick or irregular capsule that appears disrupted or focally invaded, but the findings are either due to artifactual or inadequately sampled lesion to be definitive. Factors such as tangential sectioning, poor fixation, and post-FNA fibrosis can all obscure the true capsule-tumor interface. In these cases, both WHO and CAP guidelines stress that a diagnosis of carcinoma should only be made with unequivocal evidence of invasion. If that threshold is not met, even if the findings are suspicious, the appropriate diagnosis is UMP.

Molecular testing may reveal *RAS* or *RAS*-like alterations, without the presence of high-risk mutations such as *BRAF* V600E or *BRAF*-like, which are incompatible with a UMP diagnosis.

Clinically, most UMP tumors exhibit benign or indolent behavior, like follicular adenomas or NIFTP. For this reason, lobectomy followed by clinical surveillance is typically sufficient, and completion thyroidectomy or radioactive iodine therapy is generally not recommended unless other high-risk features are present [43, 44]. Nonetheless, close follow-up is warranted due to the inherent diagnostic uncertainty. Some experts suggest that with more extensive tissue sampling or review by experienced thyroid pathologists, many UMP cases could be reclassified as either clearly benign or definitively malignant [45, 46]. In this regard, the UMP designation often reflects a cautious diagnostic approach rather than a distinct biological entity.

### Malignant neoplasms

Encapsulated thyroid carcinomas comprise a heterogeneous group of well-circumscribed malignant tumors, including *IEFVPTC, follicular and oncocytic carcinomas, medullary carcinoma*, and more aggressive forms such as *poorly differentiated* and *high-grade well-differentiated thyroid carcinomas*. Table 2 provides a summary of these various encapsulated follicular-derived entities, including the key histologic features, molecular alterations, and clinical behavior.

**Table 2:** Key Characteristics of follicular cell-derived encapsulated tumors

Entity	Growth pattern	PTC NF	Cap Inv	Vas Inv	True Pap	PB	Nec/high MA	Com Mol Alt
FA	Follicular, solid, trabecular, papillary, etc	Absent	Absent	Absent	+ ve—FA with Pap-Architecture	Absent	Absent/absent	RAS/RAS-like
OA	Solid, trabecular, follicular, etc	Focal nuclear elongation; rare IntraN grooves	Absent	Absent	Rare	Rare cases can show cytoplasmic PBs	Absent/absent	MT-DNA alteration RAS
NIFTP	Follicular, < 30% solid growth	Present	Absent	Absent	Absent	Absent	Absent/absent	RAS/RAS-like
FT-UMP	Follicular	Absent	Questionable	Absent	Absent	Absent	Absent	RAS/RAS-like
WDT-UMP	Follicular	Questionable	Questionable	Absent	Absent	Absent	Absent	RAS/RAS-like
FCA-Mi	Follicular	Absent	Present	Absent	Absent	Absent	Absent	RAS/RAS-like
FCA-AI	Follicular	Absent	Present	Present: Focal = < 4 vessels; Extensive = ≥ 4 vessels	Absent	Absent	Absent	RAS/RAS-like
EC-PTC	Papillary	Present	±	Usually absent	Present	Present	Absent	BRAF-V600e/BRAF-like
IEFVPTC-MI	Follicular	Present	Present	Absent	Absent	Usually absent	Absent	RAS/RAS-like
IEFVPTC-AI	Follicular	Present	Usually absent	Present: Focal = < 4 vessels; Extensive = ≥ 4 vessels	Absent	Usually absent	Absent	RAS/RAS-like
OCA-MI	Solid, trabecular, follicular, etc	Present	Present	Absent	Absent	Usually absent	Absent	MT-DNA alteration RAS
OCA-AI	Solid, trabecular, follicular, etc	Present	Usually absent	Present: Focal = < 4 vessels Extensive = ≥ 4 vessels	Absent	Usually absent	Absent	MT-DNA alteration RAS

*PTC *papillary thyroid carcinoma, *NF* nuclear features* Cap *capsule,* Inv *invasion,* Vas *vascular,* Pap *papillae, *PB* psammoma bodies,* Nec *necrosis,* MA *mitotic activity (defined as > 3 mitoses per 2 mm^2^),* Com Mol-Alt *common molecular alterations,* FA *follicular adenoma* OA *oncocytic adenoma,* NIFTP *non-invasive follicular thyroid neoplasm with papillary-like nuclear features,* FT-UMP *follicular tumor of uncertain malignant potential,* WDT-UMP *well-differentiated tumor of uncertain malignant potential,* EC-PTC *encapsulated classic papillary thyroid carcinoma,* IEFVPTC *invasive encapsulated follicular variant papillary  thyroid carcinoma,* MI *minimally invasive,* AI *angioinvasive,* OCA* oncocytic carcinoma

**Fig. 8:**
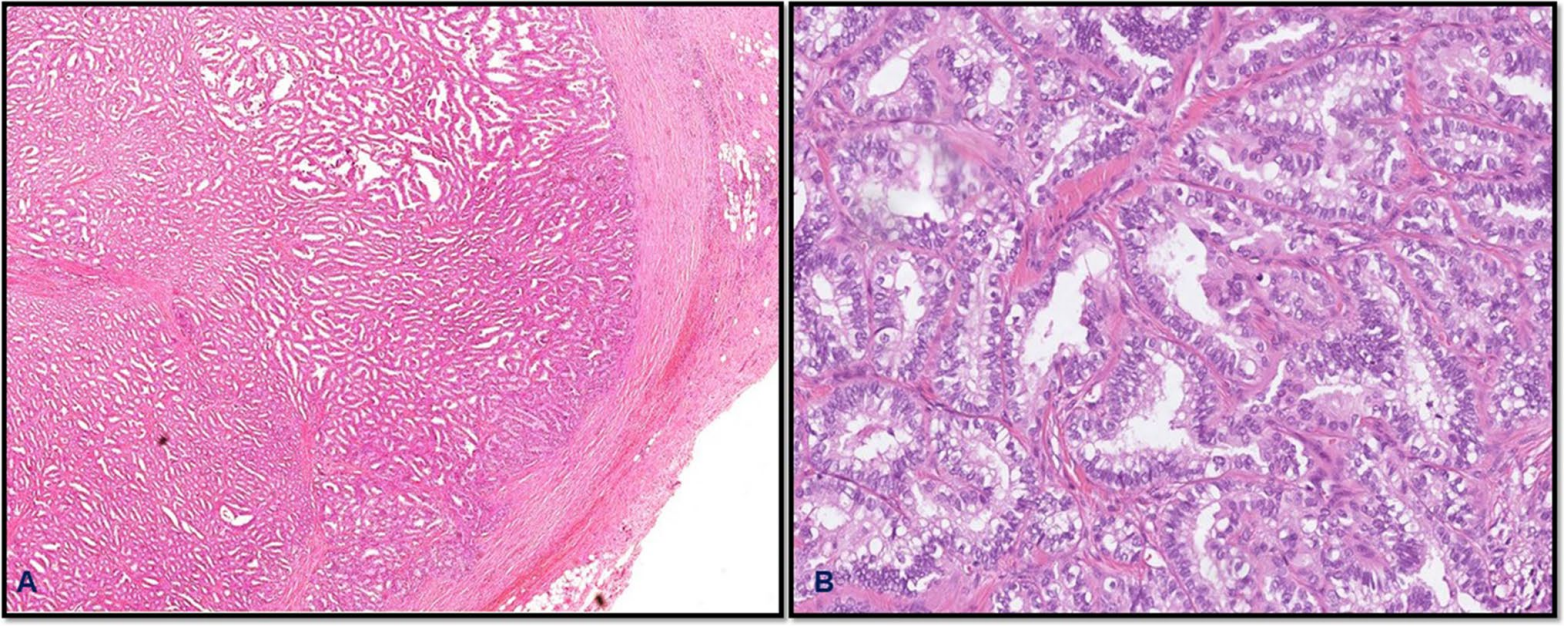
Encapsulated papillary thyroid carcinoma, classic subtype, showing well-developed tumor capsule and papillary architecture (**A**) with diagnostic typical nuclei of papillary thyroid carcinoma (**B**)

From a molecular standpoint, encapsulated thyroid carcinomas typically exhibit *RAS*-like profiles. Mutations in *NRAS* and *HRAS* are common in both IEFVPTC and follicular thyroid carcinoma (FTC), correlating with follicular architecture and a generally indolent clinical behavior. *PAX8::PPARγ* rearrangements may also be seen, particularly in FTC [20, 32]. *Oncocytic carcinomas* can share similar *RAS* pathway mutations but are also characterized by complex chromosomal losses and mitochondrial DNA mutations. In contrast, the *BRAF V600E* mutation—strongly associated with classic, tall cell, and columnar subtypes of papillary thyroid carcinoma (PTC)—is more typical of the infiltrative form of the follicular variant of PTC rather than its encapsulated counterpart [47] (Fig. 8).

**Fig. 9:**
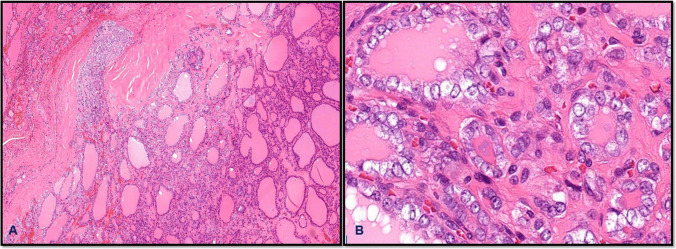
Minimally invasive encapsulated follicular variant of papillary thyroid carcinoma. Tumor shows capsular invasion (**A**) and the diagnostic nuclear features (**B**)

Histologically, *IEFVPTC* is characterized by a well-formed fibrous capsule, follicular patterned growth, and nuclear features of PTC (Fig. 9). As aforementioned, a diagnosis of carcinoma in encapsulated thyroid tumors is made only when there is definitive evidence of capsular and/or vascular invasion. In the absence of such invasion, even tumors with concerning features are classified as benign adenomas or tumors of uncertain malignant potential (UMP), depending on the degree of histologic ambiguity. FTC is a classic example of this diagnostic principle. FTC lacks the nuclear features of PTC and is typically composed of uniform follicles lined by bland epithelial cells. The distinction between FTC and follicular adenoma rests entirely on the presence of capsular and/or vascular invasion, without which a definitive diagnosis of carcinoma cannot be made. Similarly, encapsulated oncocytic carcinoma consists of oncocytes, large cells with abundant granular, eosinophilic cytoplasm, arranged in solid, trabecular, or follicular growth patterns. Like the FTC, its diagnosis also depends solely on demonstrating invasion. This is essential, as oncocytic adenomas can closely mimic carcinomas morphologically but do not exhibit invasive growth. Encapsulated thyroid carcinomas generally carry an excellent prognosis, particularly when vascular invasion is absent or minimal. For example, IEFVPTC with limited invasion behaves much like NIFTP, exhibiting a low risk of recurrence or metastasis. Similarly, minimally invasive follicular thyroid carcinoma (FTC) tends to have favorable outcomes, especially when vascular invasion is absent or limited to one to three foci [13]. However, the prognosis changes significantly with extensive vascular invasion, typically defined as involvement of four or more blood vessels. In such cases, the risk of distant metastasis increases, often necessitating more aggressive treatment, including completion thyroidectomy and consideration of radioactive iodine therapy [48].

**Fig. 10:**
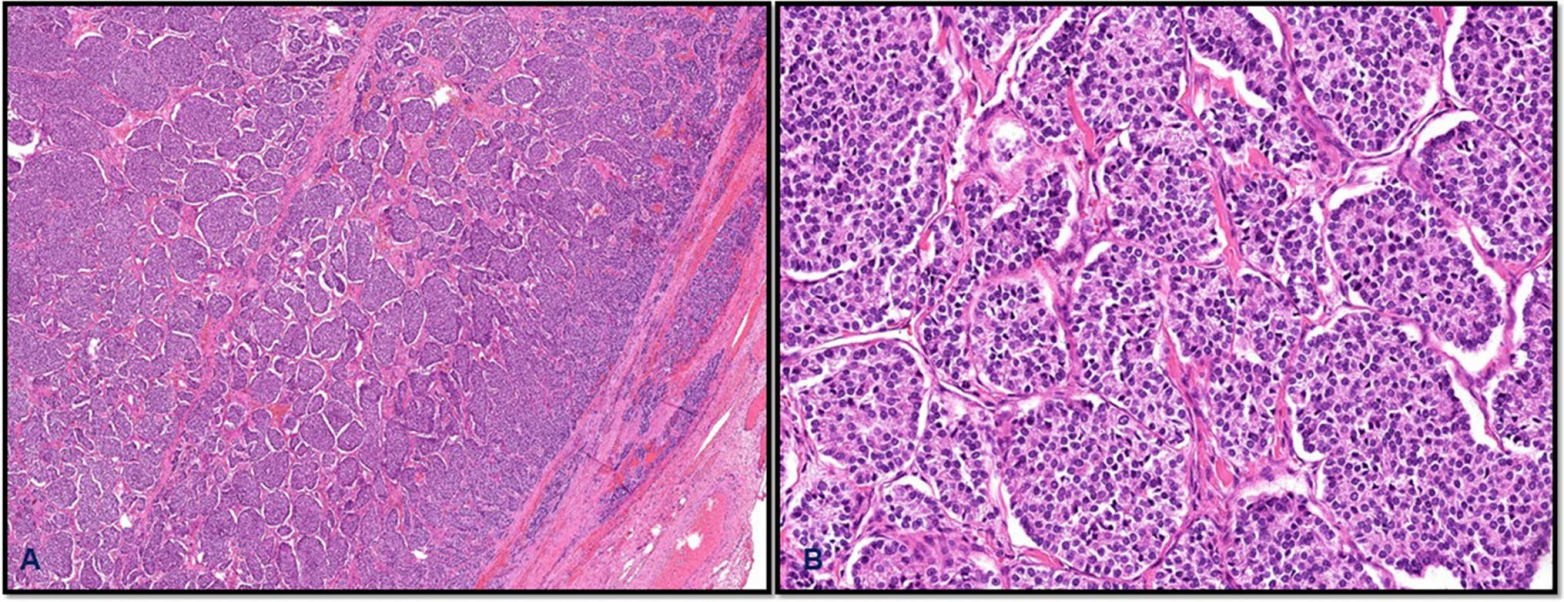
Poorly differentiated thyroid carcinoma. The tumor is encapsulated, showing capsular invasion (**A**) and insular growth pattern and lacks nuclear features of papillary thyroid carcinoma (**B**)

*High-grade differentiated thyroid carcinomas (HGDTCs) and poorly differentiated thyroid carcinomas (PDTCs)* commonly arise through progression from well-differentiated thyroid carcinomas of follicular cell origin, retaining early driver mutations such as *BRAF V600E* or *RAS*. Their mutational burden is intermediate between well-differentiated and anaplastic carcinomas. *RAS* mutations are especially prevalent in PDTCs, supporting origin from follicular carcinoma or encapsulated follicular variant papillary carcinoma, while *BRAF V600E* is more common in high-grade papillary carcinomas. Late mutations, particularly in *TP53*, *TERT* promoter, and *PI3K*/*PTEN*/*AKT* pathway genes, drive progression and are linked to aggressive behavior [49]. Macroscopically, PDTCs and HGDTCs are often large (4–6 cm), solid, and invasive, though encapsulated forms exist. PDTC is defined histologically by the Turin criteria, which require a solid/trabecular/insular growth pattern, absence of papillary nuclear features, and at least one high-grade feature (≥ 3 mitoses/2 mm^2^, necrosis, or convoluted nuclei) [50] (Fig. 10). Extrathyroidal extension and angioinvasion are common. HGDTCs, in contrast, maintain differentiated architecture (e.g., papillary or follicular patterns) but exhibit ≥ 5 mitoses/2 mm^2^ and/or tumor necrosis [51]. Most are high-grade variants of papillary carcinoma (e.g., tall cell, hobnail), while high-grade follicular and oncocytic types are less frequent. Both PDTC and HGDTC can coexist with or progress to anaplastic carcinoma. Immunohistochemically, these tumors express keratins, TTF1, PAX8, and variably thyroglobulin, with elevated Ki-67 indices (typically 10–30%) [52].

In the differential diagnosis of encapsulated tumors, non-follicular cell-derived lesions should also be considered. Table 3 provides a comprehensive summary of all encapsulated lesions that can be encountered in the thyroid gland. One such example is medullary thyroid carcinoma, a malignant neuroendocrine tumor that arises from the calcitonin-producing parafollicular C-cells and presents as a well-demarcated or encapsulated tumor, exhibiting a spindle or epithelioid morphology and nested or solid architecture. Characteristic features such as “salt and pepper” chromatin, amyloid, and elevated serum calcitonin can be helpful clues to the diagnosis, which can be confirmed with immunohistochemistry for calcitonin, CEA, and neuroendocrine markers (INSM-1, Synaptophysin, Chromogranin-A). Importantly, up to 25% of the patients with medullary thyroid carcinoma have MEN2 syndrome, and germline testing for *RET* mutations is recommended for all patients, regardless of family history [53].

**Table 3:** Differential diagnosis of encapsulated* thyroid tumors

Entity	Key histologic features	PTC-NF	Invasion	Growth pattern	Common molecular findings	Clinical behavior
FA	Encapsulated, uniform follicles	Absent	None	Micro- or normo-follicular	*RAS* mutations (sometimes)	Benign
FCA-MI	Encapsulated, follicular growth	Absent	Capsular and/or < 4 vascular	Follicular	*RAS*, *PAX8/PPARG*	Low-risk malignancy
FCA-WI	Partially encapsulated or infiltrative	Absent	Extensive capsular and/or ≥ 4 vascular invasion	Follicular, infiltrative	*RAS*, *TERT* promoter	High-risk malignancy
NIFTP	Encapsulated, no invasion, no papillae	Present (score 2–3)	None	Follicular	*RAS*, *NRAS*	Very low risk (borderline)
IEFVPTC	Encapsulated, variable papillae	Present	Capsular and/or vascular invasion	Follicular with rare papillae	*RAS*, rarely *BRAF*	Malignant
EC-PTC	Encapsulated, true papillae, psammoma bodies	Prominent	Maybe present	Papillary	*BRAF V600E*	Malignant
OA	Encapsulated, oncocytic cells, uniform	Absent	None	Solid/follicular/trabecular	Mitochondrial DNA mutations	Benign
OCA	Encapsulated or invasive, oncocytic	Absent	Present	Solid/follicular/trabecular	Mitochondrial DNA mutations, *TERT*	Malignant
PDTC (ENCAPSULATED)	Solid/trabecular/insular, necrosis and/or high mitotic index	Absent or minimal	Invasive or necrotic	Solid/trabecular	*TP53*, *TERT*, *RAS*	Aggressive
HG-WDTC (ENCAPSULATED)	Well-differentiated carcinoma morphology, necrosis, and high mitotic index	Present in PTC subtypes	Invasive	Well-differentiated carcinoma morphology	*BRAF V600e* or *RAS*, *TERT*	Aggressive
MTC (ENCAPSULATED/WELL-CIRCUMSCRIBED)	Neuroendocrine morphology, amyloid	Absent	Often not encapsulated	Solid/nested	*RET* mutations	Malignant
PN-INT**	May appear encapsulated, nests/acini	Absent	NA	Solid/acinar	*MEN1, HRPT2*	Depends on the type (adenoma vs carcinoma)
THYMOMA – INT**	Encapsulated or well-delineated mass forming jigsaw puzzle-like lobules separated by fibrotic bands	Absent	Usually absent	Resembles mediastinal thymoma- Biphasic: epithelial cells + lymphocytes	NA	Usually benign, rare cases with lymph node and lung mets reported
SETTLE**	Encapsulated, biphasic tumor: mitoses and focal necrosis are rare	Absent	Usually absent, capsular invasion is rare	spindle cells + epithelial, fascicular spindle cell areas, immature glandular structures, minimal atypia,	*KRAS, NRAS, SMYD1 (KMT3D),* or *KMT2C*	Usually indolent but may metastasize late
CMTCA**	Encapsulated, multiple lobules, squamoid morules	Rare	Both capsular and vascular invasion can be seen in a subset of cases	Mainly cribriform growth can show follicular, papillary, trabecular, and solid growth,	*Wnt/beta-catenin* with *APC* mutations in both sporadic and familial cases, *CTNNB1* mutations, *RET/PTC, PIK3CA, RAS, TERT* in aggressive tumors	

*Encapsulation does not equate to benignity; adequate sampling of the tumor and capsule interface and careful attention to capsular and vascular invasion are critical

**Immunohistochemistry is critical to distinguish these from primary thyroid tumors

*NF-PTC *nuclear features of papillary thyroid carcinoma,* FA *follicular adenoma,* NIFTP *non-invasive follicular thyroid neoplasm with papillary-like nuclear features,* IEFVPTC *invasive encapsulated follicular variant papillary carcinoma,* EC-PTC *encapsulated classic papillary thyroid carcinoma,* OA *oncocytic adenoma,* OCA *oncocytic carcinoma,* MTC *medullary thyroid carcinoma,* PN *parathyroid neoplasm,* INT *intrathyroidal,* SETTLE *spindle epithelial tumor with thymus-like elements,* CMTCA* cribriform morular thyroid carcinoma

*Cribriform morular thyroid carcinoma (CMTCA)*, formerly known as the *cribriform-morular variant of PTC*, is a rare malignant thyroid tumor with a predilection for young females. It may occur sporadically, due to somatic mutations (e.g., *APC*, *CTNNB1*, *AXIN1*, *RET*, *PIK3CA*, *KRAS*, or *TERT* promoter), or in up to 53% of cases, in association with Familial Adenomatous Polyposis (FAP) via germline *APC* mutations [54]. CMTCA typically presents as an encapsulated or well-circumscribed nodule showing diverse growth patterns: cribriform, follicular, papillary, trabecular, solid, and morular (squamoid). Angioinvasion and capsular invasion occur in about one-third of cases. Strong nuclear and cytoplasmic β-catenin staining is a diagnostic hallmark for this entity. Additionally, the tumor cells are positive for TTF-1 in the cribriform areas, while thyroglobulin and PAX8 are typically negative in both the cribriform and squamoid components of the tumor. Morular areas are positive for CDX2 and CD10 [55, 56]. Current treatment largely follows the guidelines for differentiated thyroid carcinoma. Lymph node metastases are not uncommon, particularly at the time of presentation. For FAP-associated cribriform morular thyroid carcinoma, total thyroidectomy is necessary due to the frequent presence of multicentricity and bilaterality [57].

**Fig. 11:**
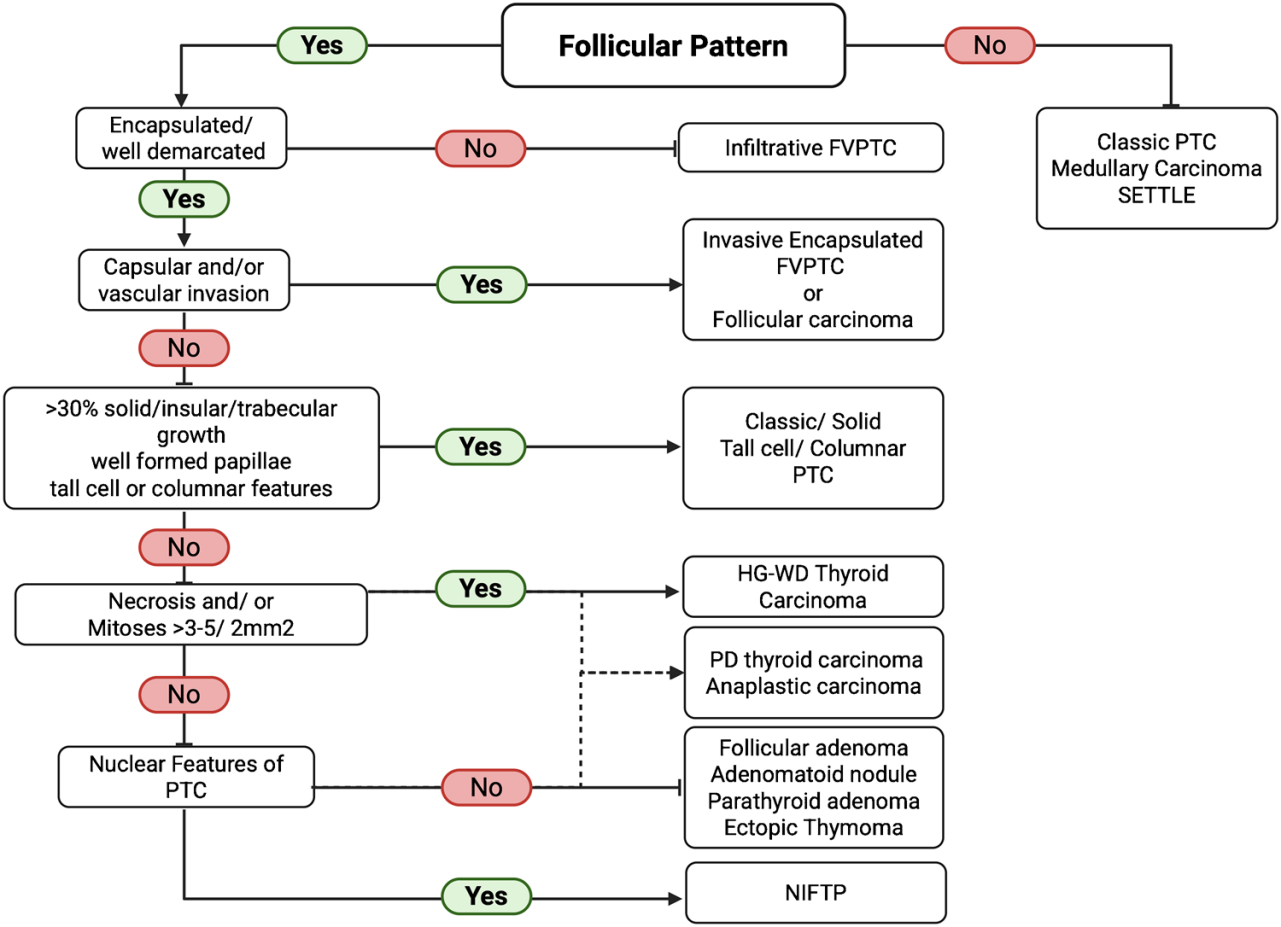
Concise diagnostic algorithm of encapsulated thyroid lesions (image created using Biorender.com)

Spindle epithelial tumor with thymus-like elements (SETTLE) is a rare malignant intrathyroidal tumor thought to originate from intrathyroidal thymic tissue. These tumors are grossly encapsulated and have a vaguely lobulated architecture with intersecting variably thick fibrous septa. Histologically, they contain spindle and glandular structures, with cleft-like spaces containing grey-blue mucin. *KRAS* and *NRAS* mutations have been reported [58–60]. Both components express pan-cytokeratin, while focal staining for myoepithelial markers (SMA and MSA) may be found in the spindle cell component. Vimentin, EMA, p40/p63, CD99, BCL2, KIT (CD117), SMARCB1 (INI1), and TLE1 can be focally positive, while CK20, thyroglobulin, calcitonin, CEA, TTF1, CD5, TdT, S100, desmin, CD34, calretinin, and WT1 should be negative. Figure 11 provides a diagnostic algorithm aid in the evaluation and classification of encapsulated lesions of the thyroid.

## Conclusions

In summary, encapsulated thyroid carcinomas form a diagnostically challenging yet clinically significant group of tumors. Because their appearance on imaging and cytology often mimics benign lesions, definitive classification requires meticulous histologic assessment and, when appropriate, molecular testing. The critical distinction between benign and malignant encapsulated lesions hinges on the unequivocal identification of capsular and/or vascular invasion, requiring comprehensive tissue sampling and strict adherence to established diagnostic criteria. As diagnostic technologies advance and molecular insights deepen, the precision and consistency of classification will continue to improve, helping pathologists and clinicians avoid both undertreatment of malignant disease and unnecessary intervention in benign conditions. Moving forward, integrating histopathologic rigor with molecular diagnostics will be essential to refining risk stratification and ensuring personalized, evidence-based management for patients with encapsulated thyroid neoplasms.
